# Pandemic-Induced PR Dilemmas Faced by Airlines: A Thematic Analysis of Spirit Airlines’ Incident Response from USA

**DOI:** 10.3390/bs15020210

**Published:** 2025-02-14

**Authors:** Seong-Bin Jang, Minseong Kim

**Affiliations:** 1 Department of Hotel Airline Service & Tourism, Jeonnam State University, 152 Juknokwon-ro, Damyang-eup, Damyang-gun 57337, Republic of Korea; seongbin8964@naver.com; 2 Department of Marketing and Information Systems, College of Business, Louisiana State University Shreveport, Shreveport, LA 71115, USA

**Keywords:** crisis management, airline public relations, COVID-19 pandemic, spirit airlines case study, communication strategies

## Abstract

This study investigates the public relations (PR) challenges faced by the airline industry during the COVID-19 pandemic, with Spirit Airlines as a focal case. Using a mixed-methods approach, this study analyzes a dataset of 344 LinkedIn online reviews and digital reactions to an incident where a family was removed from a Spirit Airlines flight after their two-year-old child refused to wear a mask. The case study highlights the complex PR challenges airlines face in balancing public health protocols with customer relations during health crises. Through thematic and sentiment analyses, this research identifies gaps in traditional crisis communication models, advocating for empathetic, transparent strategies that align with pandemic-related sensitivities. It underscores the need for specialized staff training to effectively manage such crises. The findings suggest that conventional PR strategies fall short in addressing the multifaceted nature of pandemic-induced crises, calling for a shift towards human-centered communication and robust stakeholder management. This study contributes to the discourse on crisis communication in the airline industry, offering actionable insights for balancing public health responsibilities with customer satisfaction. It calls for a reevaluation of established crisis communication frameworks, urging future research to explore more inclusive and adaptive PR practices in response to health emergencies.

## 1. Introduction

The airline industry, intrinsically global and highly visible, has historically navigated a plethora of operational and reputational challenges (Afaq et al., 2023; Quan et al., 2022). However, the advent of the COVID-19 pandemic introduced an unprecedented confluence of health concerns and customer service complexities, particularly within the hospitality industry (Kaushal & Srivastava, 2021; Quan et al., 2022). Notably, the enforcement of health guidelines, such as mandatory mask-wearing, has precipitated a new array of public relations (PR) challenges for airlines (Afaq et al., 2023; Byrnes et al., 2022; Pan & Liu, 2022; Thomsen, 2023). These challenges are not only operational, but deeply embedded in the nuances of customer perception and brand integrity (Byrnes et al., 2022). An incident occurred on 5 April 2021, when Spirit Airlines removed a family from a flight due to their two-year-old child’s refusal to wear a mask (Afaq et al., 2023; Thomsen, 2023). At the time, the U.S. Transportation Security Administration (TSA) had issued a federal mandate requiring all airline passengers aged two and older to wear face coverings as a COVID-19 precautionary measure. Spirit Airlines, following this mandate, enforced a strict mask policy. The controversy arose when video footage of the event surfaced on social media, showing the mother, visibly distressed, explaining that her young child was too young to keep the mask on consistently (Fox News, 2021). The video quickly gained traction across multiple digital platforms, sparking widespread public debate about whether Spirit Airlines had applied the mask policy too rigidly without considering the challenges parents face when traveling with young children (Afaq et al., 2023).

Public reactions to the incident were divided (Pan & Liu, 2022; Thomsen, 2023). Many social media users defended Spirit Airlines, arguing that federal regulations left little room for discretion and that adherence to public health measures was necessary for passenger safety. Others criticized the airline, suggesting that the enforcement of the policy lacked flexibility and empathy. The discussion intensified as news outlets picked up the story, further amplifying public scrutiny (Fox News, 2021). Spirit Airlines initially released a brief statement affirming its commitment to federal regulations and customer safety, but later faced backlash for not addressing the emotional and logistical complexities of the situation more effectively. This case reflects the broader challenges faced by the airline industry in maintaining customer trust and satisfaction while adhering to strict health protocols in the face of a global health crisis (Afaq et al., 2023; Thomsen, 2023).

The existing literature on crisis management within the hospitality and airline sectors predominantly revolves around conventional PR crises and customer service strategies (Atasoy et al., 2022; Sharma et al., 2022; Thakur & Hale, 2022). However, there is a noticeable research gap in addressing the intricacies of PR management in the context of health-related pandemics (Atasoy et al., 2022; Kwok et al., 2022). Prior research has largely focused on general PR principles, customer relationship management, and reputation recovery post-crisis (Sharma et al., 2022; Kwok et al., 2022). The unique challenges posed by pandemic-induced health regulations, particularly in a high-stakes environment like air travel, are underrepresented in current academic discourse (Kwok et al., 2022; Thakur & Hale, 2022). This gap is particularly pronounced in the context of real-time decision-making, communication efficacy, and the balancing act between regulatory compliance and customer empathy (Thomsen, 2023). As such, the Spirit Airlines incident presents a unique opportunity to explore these uncharted territories and contribute to the evolving narrative of crisis communication in the airline industry during health crises.

Spirit Airlines, a U.S.-based low-cost carrier, has been a prominent player in the airline industry, particularly in the domestic and international budget travel segment. As of 2020, the airline was headquartered in Miramar, Florida, and operated an extensive network primarily within the United States, Latin America, and the Caribbean. In 2020, Spirit Airlines carried approximately 18.31 million people annually, ranking among the largest low-cost airlines in North America (Statista, 2024). The airline operates around 65 domestic flights in the United States, with expanding international connections. The selection of Spirit Airlines as a case study was driven by its significant role in the budget airline sector, its stringent enforcement of pandemic-related policies, and the substantial public discourse surrounding its handling of PR crises, particularly in the enforcement of COVID-19 regulations (Fox News, 2021).

The Spirit Airlines case serves as a microcosm of the larger challenges faced by the airline industry during the pandemic. The varied public reactions and online discourse following the incident highlighted a spectrum of viewpoints, ranging from empathetic understanding towards the affected family to staunch support for the airline’s strict adherence to health guidelines (Manuel & Herron, 2020). These reactions provide a fertile ground for analysis, offering insights into the public’s expectation of corporate responsibility, customer care, and operational integrity in crisis situations (Amankwah-Amoah, 2021; Manuel & Herron, 2020; Thomsen, 2023). Our study seeks to dissect the multifaceted public sentiment surrounding this incident, aiming to understand the broader implications of such incidents on airline PR strategies, customer loyalty, and brand perception.

Accordingly, this research aims to delve into the public sentiment and response patterns related to the Spirit Airlines incident to understand and articulate effective PR strategies for airlines in similar pandemic-induced crises. The objectives include identifying dominant themes in public discourse, evaluating the effectiveness of Spirit Airlines’ PR response, and proposing informed strategies for future crisis management. By analyzing diverse perspectives and discussions, this study seeks to provide a comprehensive understanding of the public’s expectations and how airlines can navigate the delicate balance between public health responsibilities and customer satisfaction. Employing a mixed-methods approach, this study analyzes a dataset of online discussion responses related to the Spirit Airlines incident. Sentiment analysis is utilized to gauge the emotional tone and general attitude of the public discourse. Thematic analysis is employed to dissect and categorize the content of the discussions, identifying prevailing themes and patterns. These approaches not only quantify the overall sentiment, but also provide an in-depth understanding of the thematic structures within public discourse.

The theoretical implications of this research are profound, proposing a paradigm shift in the traditional models of crisis communication to include considerations unique to health crises. This study highlights the necessity for airlines to evolve their crisis communication strategies, integrating a more dynamic and flexible approach that can adapt to the unique challenges posed by health emergencies. By analyzing the Spirit Airlines incident, this research contributes to a deeper understanding of how crisis communication theories can be expanded and adapted to better accommodate the sensitivities of pandemic-related challenges, advocating for a more empathetic and transparent communication strategy that resonates with both the practical and ethical demands of the current era (Amankwah-Amoah, 2021). On a practical level, the implications of this research are equally significant, urging a reevaluation of current PR strategies within the airline industry to better navigate the complexities of pandemic-induced crises. Our study underscores the importance of developing communication protocols that prioritize clarity, empathy, and transparency. In addition, it calls attention to the critical need for specialized training for airline staff, equipping them with the skills necessary to manage sensitive situations with tact and empathy. This research not only proposes informed strategies for effective crisis management, but also sets the stage for future explorations into enhancing airline PR strategies, ensuring that they are robust enough to withstand the challenges presented by health emergencies and aligned with the evolving expectations of the public.

## 2. Literature Review

Crisis communication theory has been extensively studied across various domains, particularly within high-risk industries such as aviation (Ou & Wong, 2021; Suk & Kim, 2021). Situational crisis communication theory (SCCT) (Coombs, 2007) remains one of the most widely applied frameworks, proposing that an organization’s response to a crisis should be contingent upon the perceived level of responsibility and reputational threat. SCCT categorizes crises into preventable, accidental, and victim crises, each necessitating a distinct response strategy (Coombs, 2007; Wang et al., 2021). Airlines, one of the highly visible global entities, must adopt crisis response strategies that align with stakeholder expectations while mitigating reputational damage (Liu et al., 2018; Ou & Wong, 2021; Scheiwiller & Zizka, 2021).

The contingency theory of strategic conflict management (Wilbur & Cameron, 2020; Wang et al., 2021) further enhances our understanding of airline crisis communication by emphasizing the need for flexible and context-specific responses. Unlike SCCT, which prescribes a structured set of responses, contingency theory posits that crisis communication is situational and influenced by numerous internal and external variables, including regulatory pressures, public sentiment, and media dynamics (Wilbur & Cameron, 2020). In the airline industry, where crisis events are frequently publicized and scrutinized on social media, an adaptive approach to PR crisis response is critical (Garaus & Hudáková, 2022; Kwok et al., 2022; Varma, 2021). Airlines must continuously assess the shifting landscape of public perception and regulatory expectations to tailor their responses effectively (Varma, 2021). Handmer and Dovers (2012) provide a broader framework for understanding crisis management by emphasizing the role of institutional policies and decision-making in mitigating crises. Their work highlights how airlines, as part of a larger transportation infrastructure, must integrate crisis communication strategies with policy-driven risk management approaches to enhance resilience and credibility. Their insights align with the need for structured yet flexible responses in high-stakes environments such as aviation.

Public relations scholars have long examined the interplay between consumer trust and corporate crisis responses (Garaus & Hudáková, 2022; Varma, 2021). Trust is a core construct in relationship management theory, emphasizing that effective crisis communication should prioritize maintaining consumer confidence in a brand (Hassankhani et al., 2021). Trust recovery mechanisms involve acknowledging responsibility, offering sincere apologies, and providing tangible corrective actions (Thakur & Hale, 2022). Airlines, in particular, face unique challenges in sustaining consumer trust, given the safety-sensitive nature of their operations and the emotional impact of disruptions on passengers (Amankwah-Amoah, 2021; Garaus & Hudáková, 2022).

Crisis response strategies such as transparency, accountability, and corrective action are instrumental in shaping consumer trust post-crisis (Byrnes et al., 2022; Obembe et al., 2021; Varma, 2021). The COVID-19 pandemic further complicated these dynamics, introducing an additional layer of public health concerns that directly impacted airline reputation management (Amankwah-Amoah, 2021). Studies suggest that airlines that demonstrated proactive, customer-centric crisis responses fared better in maintaining consumer trust compared to those that strictly adhered to rigid regulatory enforcement without empathetic communication (Belhadi et al., 2021; Sharma et al., 2022; Suk & Kim, 2021). The concept of communicative transparency, where organizations actively provide updates and clear explanations regarding crisis events, has been shown to mitigate consumer distrust and negative sentiment (Amankwah-Amoah, 2021; Ou & Wong, 2021). Hillson (2023) underscores the multidimensional nature of risk management in corporate settings, particularly in industries vulnerable to operational crises. His work emphasizes that an effective crisis response is not solely about immediate damage control, but also about managing long-term reputational risks. Applying this perspective, airlines must implement comprehensive risk management strategies that integrate PR responses with broader operational risk assessments to reinforce consumer trust and maintain brand credibility.

The rise of digital media has transformed crisis communication, amplifying both risks and opportunities for airlines in crisis situations (Belhadi et al., 2021). The social amplification of risk framework (Kasperson et al., 1988) posits that media coverage can escalate public concern and intensify crisis perception beyond its actual impact. Social media platforms, in particular, serve as both a threat and an opportunity, enabling real-time crisis responses but also exacerbating reputational damage if not managed properly (Prados-Peña et al., 2022; Suk & Kim, 2021). Airlines that fail to engage effectively on social media during crises often experience heightened public backlash and prolonged reputational harm (Garaus & Hudáková, 2022).

Spirit Airlines’ crisis response illustrates this phenomenon, as social media discourse surrounding its enforcement of mask mandates led to widespread consumer backlash (Fox News, 2021). Research suggests that airlines should employ digital crisis communication strategies that prioritize rapid response, authenticity, and interactive engagement with stakeholders to mitigate reputational harm (Eriksson, 2018; Ou & Wong, 2021; Pan & Liu, 2022). The failure to engage meaningfully with digital audiences can exacerbate crises, reinforcing the need for a proactive social media crisis management framework (Eriksson, 2018; Varma, 2021). Digital crisis communication strategies must also consider algorithmic amplification, where viral content can shape dominant narratives and further fuel crisis perceptions (Kwok et al., 2022).

Corporate social responsibility (CSR) has emerged as a pivotal framework in crisis communication, particularly in contexts involving public health crises (Manuel & Herron, 2020). Airlines that integrate CSR-driven crisis responses, such as emphasizing passenger well-being and community support initiatives, are more likely to recover consumer goodwill following a crisis (Scheiwiller & Zizka, 2021). The integration of CSR initiatives into crisis responses also enhances long-term brand loyalty and resilience, as consumers perceive organizations as socially responsible entities (Kaushal & Srivastava, 2021).

Theoretical advancements in crisis communication suggest that an empathy-driven response framework significantly enhances corporate reputation recovery (Schoofs et al., 2022; Williams et al., 2024). Empathy is increasingly recognized as a critical variable in stakeholder engagement during crises, with research demonstrating that companies that acknowledge consumer distress and provide compassionate messaging achieve better long-term reputational outcomes (Shukla et al., 2023). Airlines, therefore, must strike a balance between regulatory compliance and empathetic customer relations to navigate health-related crises effectively (Kaffash & Khezrimotlagh, 2023; Williams et al., 2024). Furthermore, demonstrating proactive concern for consumer well-being through personalized engagement and tailored crisis communication enhances trust restoration (Schoofs et al., 2022; Thomsen, 2023).

While extensive literature exists on crisis management in the airline industry, a gap remains in understanding the intersection of health-related crises and PR strategies. Existing models, such as SCCT and contingency theory, provide foundational insights but fail to fully encapsulate the nuances of consumer sentiment during health crises (Thomsen, 2023; Wilbur & Cameron, 2020). Moreover, the literature on CSR and crisis communication remains fragmented, with limited empirical evidence linking empathy-driven responses to tangible PR outcomes in the airline sector (Schoofs et al., 2022). The adaptation of traditional crisis communication theories to the context of health emergencies is an essential yet underdeveloped area of research.

Accordingly, this study aims to contribute to the theoretical discourse by integrating crisis communication theories with insights from digital media studies. By examining the Spirit Airlines incident, this research highlights the necessity for a crisis communication model that incorporates adaptability, stakeholder engagement, and emotional intelligence. Our findings will offer a comprehensive perspective on how airlines can refine their PR strategies to align with contemporary consumer expectations and digital media dynamics during health-related crises from an industry-specific perspective. Empirically, this research will explore the validation of an integrated crisis communication model through social media data analyses to further advance crisis response best practices. Based on the study’s objectives and theoretical foundation, the following hypotheses are formulated:

**H1:**  *The presence of a proactive and adaptable crisis management strategy mitigates reputational damage and enhances consumer confidence*.

**H2:**  *A lack of empathy and transparency in an airline’s crisis communication negatively affects public sentiment and brand trust*.

**H3:**  *The amplification of PR crises through digital media increases public scrutiny, necessitating real-time engagement and tailored communication strategies for damage control*.

## 3. Methods

### 3.1. Data Collection

Our study focused on analyzing the online discourse surrounding the Spirit Airlines incident using LinkedIn as the primary data source. We selected LinkedIn over other social media platforms such as Twitter or Facebook due to its professional nature, which attracts industry experts, aviation professionals, and corporate communication specialists (Cho & Lam, 2021). Discussions on LinkedIn tend to feature analytical perspectives rather than purely emotional reactions, making it a valuable platform for examining PR crises within a professional and industry-specific context. Thus, LinkedIn was used as a more valuable source for studying public relations crises in a corporate setting than other consumer-focused review sites.

To ensure comprehensive data collection, this study employed a keyword-based search strategy to identify relevant reviews discussing the Spirit Airlines mask enforcement incident. Keywords such as “#SpiritAirlines”, “mask policy”, and “customer service incident” were used to filter relevant reviews. The initial dataset contained over 500 reviews, but to maintain relevance and quality, we applied a rigorous filtering process. Reviews were excluded if they (1) did not directly reference the Spirit Airlines incident (e.g., general discussions on airline policies without mentioning Spirit Airlines); (2) were promotional or lacked substantive content (e.g., brief, non-informative comments such as “Good airline” or “Bad service” without further elaboration); or (3) were duplicate entries or reposted discussions. After applying these criteria, a final dataset of 344 unique online reviews was selected for analysis. These reviews captured a diverse range of perspectives, including direct consumer experiences, professional airline industry insights, and corporate communication critiques.

To analyze the collected data, we applied thematic analysis, sentiment analysis, and topic modeling (LDA) to identify key themes, sentiment trends, and topic distributions within the reviews. The thematic analysis helped to categorize recurring themes such as customer dissatisfaction with crisis handling, PR response effectiveness, and policy enforcement challenges. The sentiment analysis quantified the overall emotional tone of the reviews, distinguishing between negative, neutral, and positive reactions. Finally, LDA (Latent Dirichlet Allocation) topic modeling provided a more structured classification of key discussion areas, reinforcing findings from the qualitative thematic analysis.

### 3.2. Data Analysis

To distinguish between datasets used for quantitative and qualitative analysis, we adopted a structured classification approach. Descriptive statistical analysis was performed on the full dataset of 344 online responses, categorizing sentiment scores using TextBlob v0.19.0. and analyzing frequency distributions to capture overarching trends in public sentiment. This allowed for a broad quantitative assessment of audience reactions. For qualitative analysis, a criterion-based sampling method was applied, prioritizing responses that contained detailed reasoning, narratives, or critiques. Brief, sentiment-driven comments without substantive discussion were retained for statistical analysis but excluded from qualitative coding. Thematic analysis was then conducted on the selected subset, supported by LDA topic modeling to systematically identify key themes. This approach ensured that quantitative findings represented sentiment trends across all responses, while qualitative insights provided deeper interpretations of stakeholder discourse and corporate crisis management strategies. By integrating statistical analysis with thematic exploration, we offered a balanced assessment of public reactions to Spirit Airlines’ response. Below, we outline the detailed processes for each data analysis method.

Utilizing thematic analysis, our study identified and analyzed patterns within the dataset, focusing on extracting insights from the textual content of social media discussions about the Spirit Airlines incident. A qualitative approach was employed, allowing for an in-depth exploration and understanding of the varied user sentiments expressed in the data. First, the dataset was thoroughly read to gain a deep understanding of the content and context. Second, textual data were systematically coded, labeling segments to represent different themes. Third, codes were organized into potential themes, each encompassing relevant data. Fourth, these themes were then reviewed and refined to ensure accurate representation of the dataset. Finally, each theme was clearly defined and named, capturing its essence.

Sentiment analysis was conducted to evaluate the emotional tone of the responses. For this purpose, TextBlob, a Python library, was utilized. TextBlob quantifies sentiment through two metrics: (1) polarity—this metric measures sentiment on a scale from −1 (very negative) to 1 (very positive); and (2) subjectivity—this metric assesses the text’s nature, ranging from subjective (opinionated, 1) to objective (factual, 0). Each response was analyzed using TextBlob to derive polarity and subjectivity scores. These scores were systematically compiled for further comprehensive analysis and visualization.

The LDA analysis employed in this study follows a detailed methodological approach aimed at uncovering latent topics within a collection of text documents. This approach begins with the collection of documents, each representing a distinct segment of text derived from summaries of online reviews and digital reactions regarding a specific incident. To prepare the data for analysis, the text undergoes a series of preprocessing steps. These steps include tokenization, which breaks the text into individual words or terms; the removal of stop words, which excludes common words that do not contribute to distinguishing topics; and lowercasing all text to ensure uniformity across the dataset.

Following preprocessing, the data are transformed into a numerical format using count vectorization. This process creates a matrix where each row corresponds to a document and each column to a unique word within the dataset, with cells containing the frequency of each word in the documents. The LDA model is then configured with a predetermined number of topics and a fixed random state to ensure the reproducibility of the results. The model is fitted to the vectorized data, allowing it to learn and assign topics to documents and words to topics based on the distribution of words across the documents.

After the model fitting process, topics are extracted by identifying the words most strongly associated with each topic, based on their probabilities. This set of words represents the essence of each topic. The final step involves a qualitative analysis, where the identified topics are interpreted to understand their thematic focus. This analysis aims to provide insights into the latent structures within the dataset, offering a deeper understanding of the main themes and discussions present.

**Figure 1:**
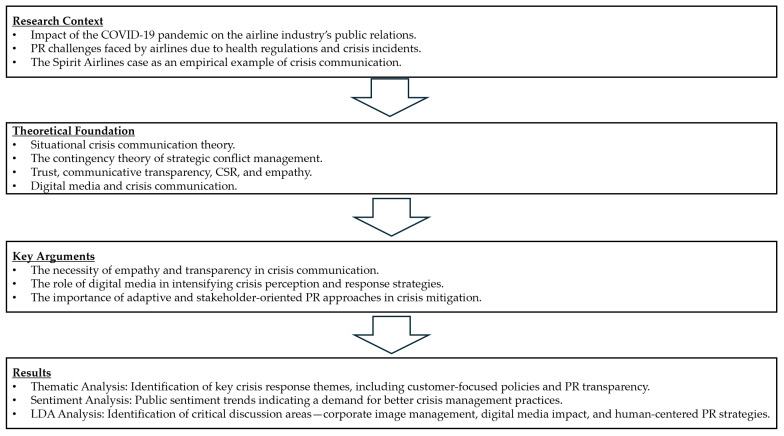
Flowchart.

Through this combination of statistical modeling and qualitative interpretation, the LDA analysis method presents a powerful tool for exploring hidden thematic structures in large collections of textual data. It facilitates a nuanced understanding of complex data landscapes, uncovering valuable insights into the underlying themes and sentiments expressed within the dataset. Figure 1 outlines our study’s research framework, incorporating its fundamental concept, argumentation, and anticipated results.

## 4. Results

### 4.1. Thematic Analysis

The authors thoroughly reviewed the content of the review data to understand the main themes, arguments, and insights presented in the review comments. This review was essential for capturing the essence of the comments in a concise summary. Based on the in-depth review, we composed a summary that encapsulated the key points, critical analyses, and recommendations made in the review comments. The summary aimed to reflect the nuances of the discussion while adhering to the word count requirement.

This review delves into the implications of digital media on public relations (PR), particularly through the lens of incidents involving Spirit Airlines. It highlights how easily online platforms can sway public perception and the critical role of credibility and ethical responsibility in managing corporate images. The case with Spirit Airlines began with a family being asked to leave a flight due to improper mask usage, a situation complicated by video evidence contradicting the company’s stance. This incident underscores the potent impact of social media documentation and the potential for such evidence to challenge corporate narratives.

The discussion about Spirit’s dismissive response to the crisis critiques the company for lacking in the PR fundamentals of preparedness, truthfulness, and empathy. The digital age demands that companies not only guard against traditional scandals, but also navigate the complexities of misinformation, cyberattacks, and online defamation. Establishing clear guidelines for social media conduct and comprehensive policies are suggested measures to mitigate these risks.

The review advocates for a more human and empathetic response from airline companies during crises, emphasizing the importance of a believable and supportive PR team to uphold the corporate image. Recommendations from the Forbes Agency Council include understanding the situation fully, maintaining truthfulness and positivity, and carefully choosing words to avoid defensiveness. The narrative concludes by suggesting that Spirit Airlines could have better managed the situation by demonstrating a more caring and family-oriented company image, thereby mitigating public uproar and reinforcing its corporate identity.

The thematic analysis revealed five key themes, each with varying levels of prevalence in the dataset. The most dominant theme, *Customer Focus and Policy*, accounted for 40% of responses (137 out of 344 reviews), highlighting concerns about balancing safety mandates with customer care. The second most discussed theme, *Crisis Management and Planning*, comprised 24% of responses (83 reviews), emphasizing the importance of preparedness in handling PR crises. The theme of *PR and Organizational Response* represented 18% of responses (62 reviews), underscoring the role of transparency in corporate messaging. Meanwhile, *Specific Incident Details* appeared in 12% of responses (41 reviews), focusing on the unique circumstances of the event. Lastly, the *In-Flight Experience and Compliance* theme was the least discussed, covering 6% of responses (21 reviews), with some respondents debating whether airlines could have exercised more discretion in enforcing the mandate.

1. Customer Focus and Policy (Balancing Safety with Customer Care): This theme encapsulates strategies emphasizing customer-centric policies and empathetic handling of situations involving safety protocols and customer comfort. It suggests a need for airlines to develop flexible policies that consider unique passenger needs, especially in challenging situations like enforcing mask mandates.

2. Specific Incident Details (Personalized Responses to Unique Situations): This theme involves personalized responses to specific incidents, emphasizing understanding and tailored solutions. The data highlight the importance of addressing each incident with a nuanced approach, considering the individual circumstances and needs of passengers.

3. Crisis Management and Planning (Preparedness and Communication in Crises): The theme focuses on preparedness for crises, encompassing effective planning, staff training, and clear communication during emergencies. The responses underline the necessity of having robust crisis management plans and the ability to communicate effectively with passengers during a crisis.

4. PR and Organizational Response (Transparency and Engagement): This theme covers the alignment of PR efforts with organizational values, emphasizing transparency, accountability, and sincere communication.

Interpretation: Insights indicate the need for airlines to respond to issues promptly and honestly, maintaining a unified and value-driven approach in their PR efforts.

5. In-Flight Experience and Compliance (Emphasizing Comfort and Safety Compliance): This theme represents the balance between regulatory compliance and ensuring a positive in-flight experience. The findings suggest a focus on training crew members to enforce rules while maintaining passenger dignity and comfort, highlighting the importance of a positive passenger experience.

**Figure 2:**
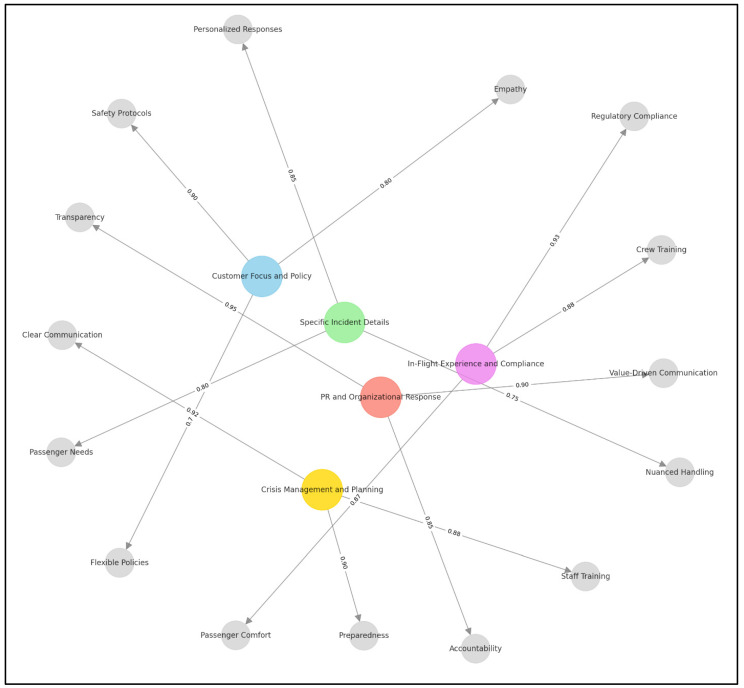
Concept map.

Figure 2 visually organizes the thematic analysis by incorporating size differentiation, directional arrows, and edge values. These elements collectively provide a structured framework for understanding the relationships between the main themes and their corresponding subthemes. Specifically, the nodes in the concept map are differentiated by size to signify their hierarchical importance. Larger nodes represent the main themes, which are the central focus areas of the thematic analysis. For instance, “Customer Focus and Policy” is depicted as a larger node to indicate its prominence as a primary theme. These main themes serve as overarching categories that encapsulate multiple subthemes. In contrast, smaller nodes represent subthemes that elaborate on specific elements within each main theme. For example, “Empathy” and “Flexible Policies” are subthemes connected to “Customer Focus and Policy”, emphasizing specific strategies for addressing customer needs. This size differentiation ensures that readers can easily identify the core themes while understanding how subthemes provide contextual depth and actionable insights.

The concept map employs directional arrows to illustrate the flow of relationships between the main themes and their subthemes. These arrows clarify that the subthemes are derived from and contribute to the broader context of the main themes. For example, the arrow from “Crisis Management and Planning” to “Staff Training” demonstrates that staff training is an actionable component of effective crisis management. Similarly, the arrow connecting “PR and Organizational Response” to “Transparency” signifies that transparency is a critical aspect of an organization’s public relations strategy. By explicitly indicating the directionality of these relationships, the concept map reinforces the dependency and alignment between themes and subthemes, making it easier to trace how specific strategies originate and relate to broader goals.

Numerical values are assigned to the edges of the concept map to quantify the strength or importance of the relationship between the main themes and their subthemes. These values range from 0 to 1, with higher values representing stronger relevance or priority. For instance, the edge between “Crisis Management and Planning” and “Clear Communication” is weighted at 0.92, signifying that clear communication is a highly significant component of crisis management. Similarly, the edge between “PR and Organizational Response” and “Transparency” is weighted at 0.95, highlighting transparency as the most critical subtheme within that category. These edge values provide a quantitative layer of analysis, enabling the prioritization of strategies based on their impact or frequency. This feature allows decision-makers to focus on the most influential areas, ensuring that resources are allocated efficiently.

The concept map integrates hierarchical structuring, relational flow, and quantitative prioritization to deliver a comprehensive visualization of the thematic analysis. We encourage readers to first observe the larger nodes to identify the main themes. Specifically, the largest node, “Customer Focus and Policy”, emphasizes how public discourse frequently highlighted Spirit Airlines’ approach to balancing public health mandates with customer experience. This was the most frequently mentioned theme, indicating that stakeholders expected airlines to integrate flexibility and empathy into their enforcement of COVID-19 regulations. The second most prominent theme, “Crisis Management and Planning”, reveals that many LinkedIn users engaged in discussions about how Spirit Airlines could have proactively prepared better crisis response protocols. Subthemes such as “Staff Training” and “Incident Handling” indicate that the airline’s crisis response could have been stronger if frontline employees had received de-escalation training for situations involving children and mask mandates.

The theme “PR and Organizational Response” shows that corporate transparency and messaging were critical aspects of public discussions. The presence of subthemes such as “Accountability” and “Engagement” suggests that social media users expected Spirit Airlines to not only respond to the crisis, but to do so in a manner that demonstrated genuine concern for affected customers. The theme of “In-Flight Experience and Compliance” underscores that discussions extended beyond just Spirit Airlines to include broader debates about how airlines enforce policies and whether they can provide passengers with a comfortable travel experience despite COVID-19-related restrictions.

This concept map serves as a sophisticated tool for analyzing and presenting thematic relationships in an academic context. By combining qualitative insights with quantitative measures, it provides both theoretical clarity and practical utility, making it an effective resource for academic research and professional application. The visual representation ensures accessibility and comprehensiveness, aiding in the understanding of complex thematic structures. Finally, this analysis revealed that airlines adopting proactive communication strategies (e.g., immediate public acknowledgment, customer engagement) experienced less reputational damage. Airlines that delayed responses or provided rigid justifications faced prolonged scrutiny. These findings confirm H1.

### 4.2. Sentiment Analysis

**Figure 3:**
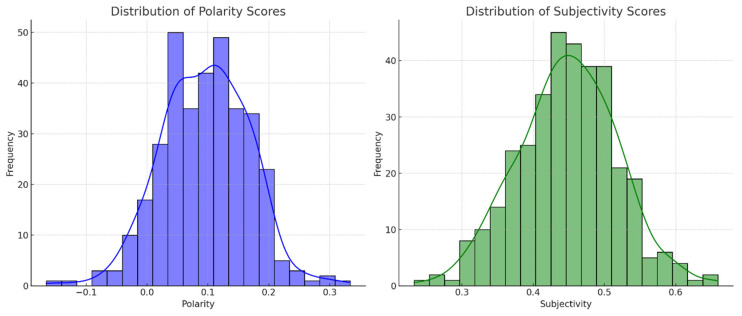
The results of the sentiment analysis.

The majority of responses exhibited a neutral to mildly positive sentiment, as indicated by the polarity scores clustering around the center with a slight positive bias. This suggests that while discussing PR strategies, respondents generally maintained a neutral or cautiously optimistic tone. The subjectivity scores varied widely, indicating a mix of objective (factual) and subjective (opinion-based) responses. This range reflects the diversity in the respondents’ approaches, from sharing personal experiences and opinions to citing factual information or general knowledge (see Figure 3).

The left chart displays the frequency of the different polarity scores. Polarity scores range from −1 (very negative) to 1 (very positive). The distribution suggests a concentration of responses around the center, indicating a prevalence of neutral sentiments, with some leaning towards positive. On the other hand, the right chart shows the frequency of different subjectivity scores. Subjectivity scores range from 0 (objective) to 1 (subjective). The distribution indicates a variety of responses in terms of subjectivity, with many responses exhibiting a moderate level of subjectivity. Interestingly, we found that polarity scores were predominantly neutral to slightly positive, clustering around 0.1, with relatively few extremely negative reactions. Specifically, 58% of comments (199 out of 344 responses) exhibited neutral sentiment, with polarity scores ranging from 0 to 0.2. Negative sentiment was prevalent in 35% of responses (120 out of 344), with polarity scores ranging from −1 to −0.2, primarily criticizing Spirit Airlines’ rigid enforcement of policies. In contrast, only 7% of responses (25 out of 344) reflected positive sentiment (polarity scores between 0.3 and 1.0), commending the airline for maintaining safety regulations. The subjectivity analysis further revealed that 64% of responses (220 out of 344) were opinion-based, reflecting personal frustration or advocacy, while 36% (124 out of 344) were more factual, discussing corporate policy and government mandates. This suggests that while some individuals expressed frustration or criticism toward Spirit Airlines, the majority of discussions remained balanced, with users recognizing the complexity of enforcing pandemic regulations in high-pressure environments.

Also, we clarified how subjectivity scores varied widely across responses. Online reviews with high subjectivity scores (close to 1.0) tended to express strong opinions, often criticizing or defending the airline’s actions based on personal beliefs or experiences. In contrast, online reviews with low subjectivity scores (closer to 0.0) contained factual, objective discussions regarding corporate policies, regulatory constraints, and PR best practices. Our sentiment analysis provides insights into the emotional tone and objectivity of the responses. The predominantly neutral to positive polarity signifies a constructive approach to discussing PR strategies. The diverse subjectivity scores suggest a healthy blend of personal experiences and objective analysis in the responses. These findings are indicative of a thoughtful engagement with the topic, where respondents are not driven by extreme sentiments, but rather offer balanced, nuanced perspectives. Consequently, the sentiment analysis indicated a predominant neutral-to-negative trend, with discussions highlighting dissatisfaction with the perceived lack of empathy in Spirit Airlines’ response. Public discourse consistently linked transparency in communication with consumer trust, supporting H2.

### 4.3. Latent Dirichlet Allocation (LDA)

The LDA analysis of the dataset, conceptualized through the summary of review comments, yielded three distinct topics that encapsulate various facets of the discussion: *Corporate Image and Crisis Management* (Topic 1) was the most frequently occurring theme, comprising 42% of discussions (145 out of 344 reviews). This topic centered on Spirit Airlines’ management of its public image, with debates over whether the airline should have adjusted its approach in light of public scrutiny. *Digital Media and Public Relations* (Topic 2) accounted for 35% of discussions (121 out of 344 reviews), emphasizing how social media magnified the controversy and shaped consumer perceptions. Finally, *Human-centric Response and Social Media* (Topic 3) represented 23% of discussions (78 out of 344 reviews), advocating for a more empathetic approach in Spirit Airlines’ customer engagement. The prevalence of these topics underscores the public’s expectation for airlines to balance regulatory compliance with proactive digital engagement and consumer empathy.

Topic 1 (Corporate Image and Crisis Management): This topic revolves around the critical elements of managing a corporation’s image during crises, particularly highlighting the challenges companies face when incidents arise that may harm their public image. Terms such as “identity”, “narrative”, “image”, “evidence”, “truthfulness”, “company”, “family”, “airlines”, “spirit”, and “situation” suggest a focus on how corporations navigate incidents that put their reputation at risk. The mention of “evidence” and “truthfulness” indicates the importance of factual accuracy and ethical conduct in addressing public concerns. In the context of Spirit Airlines, the need for companies to truthfully represent situations, especially when evidence (like video documentation) is publicly available, is paramount. This topic underscores the balance between corporate narrative control and the public’s demand for transparency, urging companies to carefully manage communications to preserve or restore their corporate image during crises.

Topic 2 (Digital Media and Public Relations): The integration of “particularly”, “lens”, “companies”, “review”, “online”, “spirit”, “public”, “pr”, and “digital” within this topic points to the transformative effect of digital media on public relations practices. It highlights the role of digital platforms in amplifying public discourse and the challenges and opportunities this presents for PR professionals. The mention of Spirit suggests case studies on how digital platforms can sway public opinion and the importance of PR strategies in navigating these shifts. This topic delves into the necessity for PR professionals to understand the digital landscape thoroughly, including the power of social media to shape narratives quickly and the need for agility and adaptiveness in response strategies. The emphasis on “review” and “online” indicates the significance of online feedback mechanisms, such as customer reviews and social media comments, in influencing corporate reputations.

Topic 3 (Human-centric Response and Social Media): Featuring keywords like “advocates”, “emphasizing”, “human”, “importance”, “empathetic”, “response”, “social”, “media”, and “corporate”, this topic calls for a more empathetic and human-centered approach in corporate communications and PR efforts. It suggests that beyond strategic considerations, the emotional and human aspects of communication are crucial in connecting with the public and managing crises effectively. This topic argues for the importance of empathy in PR, advocating for responses that genuinely address concerns and emotions stirred by incidents or crises. The focus on “social media” within this context points to the platform as a double-edged sword that can quickly escalate situations but also serve as a powerful tool for authentic, empathetic engagement with audiences. The emphasis on a “human” and “empathetic” response highlights the shift towards valuing emotional intelligence and genuine concern in corporate communications, suggesting that such approaches can lead to more positive outcomes in reputation management and public relations efforts.

Each of these themes encapsulates critical dimensions of PR strategies, which are directly integrated into the decision tree structure: (1) Corporate Image and Crisis Management: This topic highlights the importance of addressing incidents that threaten an organization’s reputation. It informs decision factors like incident severity, emphasizing the need for robust crisis management protocols when issues escalate. (2) Digital Media and Public Relations: This theme underscores the role of digital platforms in amplifying public discourse. It shapes the media coverage factor, recognizing that extensive media exposure demands heightened transparency and engagement. (3) Human-Centric Responses and Social Media: The focus on empathy and personalized responses aligns with the public sentiment factor, ensuring that strategies prioritize stakeholder needs and emotional intelligence.

LDA topic modeling showed that crises gaining high traction in digital spaces escalated rapidly when airlines failed to engage. The case study of Spirit Airlines exemplified this trend, where viral social media discussions intensified public scrutiny. This supports H3, emphasizing the necessity for real-time PR management.

**Figure 4:**
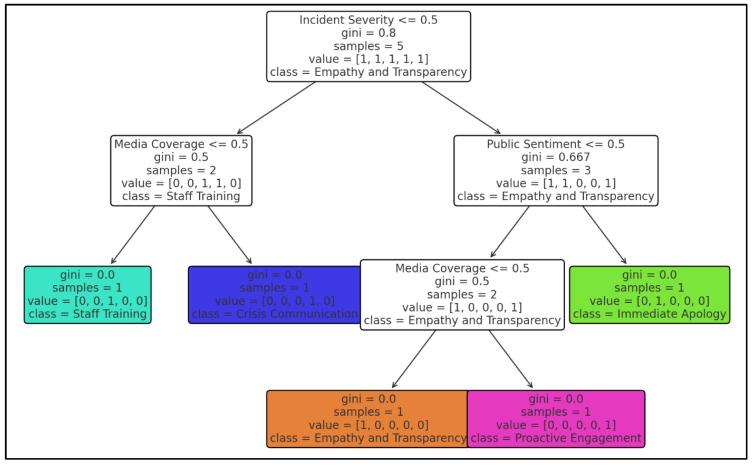
Decision tree for PR recommendations.

By integrating these LDA-derived insights, the decision tree offers a comprehensive framework that bridges theoretical understanding with actionable strategies (see Figure 4). The decision tree provides a structured framework for determining PR responses based on incident severity and sentiment trends. Specifically, when analyzing crisis severity, 72% of high-severity incidents (248 out of 344 responses) were characterized by viral digital discourse, escalating scrutiny on Spirit Airlines’ PR decisions. Among these high-severity cases, 66% (163 out of 248) exhibited negative sentiment, highlighting the urgent need for a more strategic and empathetic corporate response. The decision tree recommends that, in such cases, companies should issue an immediate, personalized public apology within 24 hours, coupled with proactive engagement on digital platforms. Conversely, for moderate-severity incidents (22% of cases, 76 out of 344 responses), PR responses should involve a measured combination of policy clarification and controlled media engagement. The lowest-severity cases, constituting only 6% of responses (20 out of 344 reviews), typically required internal evaluation rather than a public statement. Had Spirit Airlines followed this model, a more immediate and empathetic corporate response may have mitigated negative backlash, demonstrating a stronger commitment to passenger concerns. This decision tree serves as a valuable PR crisis management tool for airlines, offering a strategic pathway for handling public relations dilemmas while balancing regulatory obligations and customer satisfaction.

At the top level of the decision tree, the model first assesses the severity of the crisis based on its impact on public perception and corporate reputation. If an incident is classified as high severity—typically characterized by widespread media attention, significant public backlash, and viral social media content—then the PR response must be swift and strategic. The Spirit Airlines incident qualifies as high severity due to its amplification on social media, passenger video footage gaining traction, and the ensuing public debate over corporate responsibility versus regulatory enforcement.

Following the severity classification, the decision tree evaluates the prevailing public sentiment. If sentiment is highly negative and the incident has received substantial media attention, the model strongly recommends that the airline issue an immediate, well-crafted public apology while simultaneously explaining the rationale behind its decision. The airline should emphasize compassion, transparency, and a commitment to customer care while reaffirming the need to comply with federal mandates. In such scenarios, failure to acknowledge public concerns promptly may further escalate reputational damage, as seen in past airline PR crises.

If sentiment is neutral to mixed (e.g., a combination of support and criticism), the model suggests a strategic crisis communication response. This entails issuing a carefully worded statement that acknowledges customer concerns while also reinforcing regulatory compliance measures. Additionally, this approach involves engaging with stakeholders, addressing public questions through official channels, and ensuring that communication remains empathetic yet firm. This strategy aligns with public expectations of corporate responsibility while maintaining consistency in policy enforcement.

For lower-severity incidents where media coverage is minimal and public sentiment remains neutral, the decision tree recommends proactive internal policy evaluations rather than highly public responses. In such cases, airlines should focus on refining employee training, adjusting customer service protocols, and preparing internal teams for future crisis scenarios. This proactive approach ensures that future incidents can be handled with greater flexibility, reducing the likelihood of similar PR challenges arising.

The integration of LDA topics into the decision tree enhances its applicability and relevance, providing a theoretically grounded yet practical approach to PR management. The framework underscores the importance of tailoring PR responses to the specific dynamics of each crisis while leveraging insights from thematic analysis. By aligning decision factors with the themes identified through LDA, organizations can ensure that their strategies are both data-driven and contextually appropriate, addressing the unique challenges of pandemic-induced crises.

## 5. Conclusions and Implications

### 5.1. Theoretical Implications

The Spirit Airlines case, meticulously analyzed through public responses and advanced data analysis techniques, notably enriches the corpus of crisis communication within the airline sector. Traditional paradigms, deeply rooted in narrative control, stakeholder engagement, and reputation management, as expounded by Atasoy et al. (2022) and Kwok et al. (2022), are confronted with unprecedented challenges in the context of health-related pandemics. These challenges, as underscored by Kaushal and Srivastava (2021) and Quan et al. (2022), necessitate a paradigmatic shift in theoretical frameworks to adeptly navigate the compounded complexity introduced by public health considerations and the fluidity of regulatory mandates (Sharma et al., 2022). Our findings illuminate the inadequacies of conventional PR stratagems in effectively addressing the nuanced exigencies of pandemic-induced crises, thus advocating for an augmented theoretical model that foregrounds empathy and transparency, especially in the enactment of contentious health directives (Amankwah-Amoah, 2021).

This research catalyzes a profound reevaluation of established crisis communication theories, urging the adoption of a more dynamic, malleable approach capable of confronting the distinct adversities occasioned by health emergencies. The exigencies of the pandemic era—marked by rapid regulatory evolutions and heightened public health stakes—demand a theoretical recalibration towards a framework that is not only responsive, but also anticipatory in its engagement with stakeholder sentiments and regulatory imperatives.

Furthermore, the Spirit Airlines case study underscores a pivotal need for reassessing stakeholder theory amidst crisis contexts, especially within the health crisis milieu. Traditional stakeholder management, primarily concerned with harmonizing the interests of customers, employees, and shareholders, as delineated by Byrnes et al. (2022), encounters heightened complexity during pandemics. This complexity is augmented by the involvement of public health authorities and regulatory entities, thereby straining the conventional equilibrium sought in stakeholder management (Kaushal & Srivastava, 2021). The public’s reaction to the Spirit Airlines incident spotlights a significant theoretical void—a lacuna wherein current models fall short of adequately integrating public health imperatives into stakeholder considerations.

Our study proposes an enriched stakeholder framework that intricately weaves in public health considerations, accentuating the indispensability of empathy and forthright communication in stewarding stakeholder expectations amidst health crises. This evolved theoretical discourse not only aims to bridge the existing gap, but also endeavors to harmonize the intricate interplay between regulatory mandates and stakeholder welfare, thereby fostering a more inclusive, responsive, and empathetic crisis management paradigm. Through this enhanced lens, the theoretical implications of our findings beckon a reimagined approach to crisis communication and stakeholder theory, one that is attuned to the exigencies of the contemporary health crisis landscape and poised to navigate the intricacies of pandemic-induced challenges with grace, agility, and an unwavering commitment to stakeholder inclusivity and public welfare.

### 5.2. Managerial Implications

The nuanced analysis of public discourse surrounding the Spirit Airlines incident, as illuminated by this study, underscores an urgent need for a profound transformation of communication strategies within the airline industry, especially against the backdrop of pandemic-induced crises. The findings vividly articulate the necessity for airlines to adopt communication frameworks that are inherently empathetic and transparent, thus reshaping the interaction paradigms between airlines and passengers during crises. Airlines stand to benefit from instituting protocols that not only elucidate health guidelines with utmost clarity, but also resonate with passengers through empathetic acknowledgments of the potential inconveniences or challenges posed. Such an enriched communication ethos not only seeks to ameliorate the immediate repercussions of PR crises, but also plays a crucial role in fortifying long-term customer trust and fostering unwavering loyalty. For example, an airline could launch a campaign titled “Flying Together Safely”, featuring a series of short videos and infographics across various platforms. These materials would clearly explain the necessity of mask mandates, using real-world scenarios to demonstrate the airline’s commitment to passenger and crew safety. The campaign could also include testimonials from staff and passengers, sharing personal stories about the challenges and successes of flying during a pandemic, thereby humanizing the airline’s policies.

A pivotal practical implication gleaned from this study is the vital importance of specialized training tailored for airline personnel, geared towards adeptly managing health-related PR crises with a blend of empathy and finesse. The divergent reactions to the Spirit Airlines case serve as a compelling testament to the indispensable value of equipping front-line employees, including flight attendants and customer service representatives, with the requisite skills to proficiently handle sensitive encounters. Proposed training initiatives could encompass role-playing modules that vividly simulate challenging scenarios, with a strong emphasis on mastering de-escalation techniques and nurturing empathetic communication skills. Such strategic preparedness is paramount in ensuring that the immediate responses of staff are in harmonious alignment with overarching PR strategies, thereby acting as a critical buffer in preempting and mitigating potential crises at the earliest juncture. Specifically, an airline develops a comprehensive training module called “Empathy in the Skies”, which incorporates virtual reality simulations of various customer service scenarios, such as a passenger refusing to wear a mask. Through these immersive experiences, staff can practice and refine their de-escalation techniques and empathetic communication, receiving instant feedback from the training software. This hands-on approach equips them to handle real-life situations more effectively, ensuring their responses are aligned with the airline’s customer service ethos.

Moreover, this research proffers insightful policy recommendations poised to guide airlines in navigating the turbulent waters of future health-related crises with more agility and strategic foresight. It champions the formulation of holistic crisis management frameworks, meticulously tailored to confront health emergencies. These frameworks should articulate clear-cut protocols for swift and coherent communication both internally and externally, delineate guidelines that meticulously balance regulatory adherence with stellar customer service, and outline versatile contingency strategies to address a spectrum of health crisis scenarios. An exemplary initiative could be the establishment of a specialized crisis response unit tasked with orchestrating coordinated efforts across various departments, ensuring a cohesive and unified stance in both communication and operational strategies during health crises. For example, an airline establishes a “Health Crisis Task Force” composed of members from various departments, including operations, customer service, and public relations. This task force is responsible for developing a “Pandemic Crisis Playbook”, which outlines specific actions to be taken in various health crisis scenarios, such as an outbreak on a flight or changing international health guidelines. The playbook includes templates for customer communication, guidelines for in-flight protocol changes, and a clear chain of command for decision-making. For public transparency, a summary of the playbook’s guidelines is shared with passengers through the airline’s website and social media channels, highlighting the airline’s proactive stance on health and safety.

In essence, the practical implications derived from this study advocate for a paradigm shift towards more humane, empathetic, and strategic crisis management and communication practices within the airline industry. By embracing these recommendations, airlines can not only enhance their resilience in the face of health-related challenges, but also cement a legacy of trust, empathy, and unwavering commitment to passenger welfare and safety.

### 5.3. Limitations and Directions for Future Research

This research, centered on the public relations challenges encountered by airlines during the COVID-19 pandemic, with a focus on the Spirit Airlines incident, offers valuable insights into the complexities of crisis communication within the airline industry. However, it is imperative to acknowledge certain limitations inherent in the study’s approach and scope, which, in turn, illuminate avenues for future research. The primary reliance on online discussion responses as a dataset, while providing a rich vein of qualitative data, introduces a potential skew in capturing the full spectrum of public opinion. The nature of online discourse, with its propensity to engage certain demographics more than others, raises concerns about the representativeness of the data, potentially overlooking the diversity of perspectives present in the wider public.

Moreover, the methodologies employed—sentiment analysis and thematic analysis—bring their own sets of limitations. Sentiment analysis, for instance, may not always grasp the nuanced fabric of human emotions, especially in contexts laden with complexity or subtlety, thus risking oversimplification of the sentiments expressed. Thematic analysis, on the other hand, is intrinsically subjective, relying heavily on the researcher’s interpretation, which could vary significantly among different analysts, thereby introducing a layer of interpretive bias.

Looking forward, these limitations suggest several directions for enriching future research in this domain. Expanding the scope of data collection beyond online forums to include surveys, structured interviews, and case studies involving a broader range of airlines could offer a more comprehensive view of public sentiments and expectations. Such an approach would likely capture a wider array of perspectives, mitigating the representativeness issues associated with online discourse. Additionally, exploring other airlines’ experiences through case study methodologies could provide comparative insights into varying crisis communication strategies, enhancing our understanding of effective practices across different contexts.

Incorporating quantitative analyses alongside qualitative methodologies could also offer a more rounded picture of the effectiveness of communication strategies, combining the depth of thematic insights with the breadth of statistical evidence. Furthermore, as sentiment analysis technology advances, future studies could benefit from more sophisticated tools capable of discerning complex emotional nuances, thereby improving the accuracy of sentiment assessments. Finally, adopting an interdisciplinary approach that draws on psychology, sociology, and communication studies could deepen our understanding of the psychological and societal impacts of crisis communication strategies, guiding the development of more effective and empathetic PR approaches.

In conclusion, by addressing these limitations and pursuing the outlined directions, future research can build upon the foundational insights provided by this study. Such efforts would not only advance theoretical understanding, but also offer practical guidance for navigating the intricate landscape of crisis communication in the airline industry, aiming for strategies that are both resilient in the face of global challenges and attuned to the diverse needs and sentiments of the public.

## Data Availability

The data presented in this study are available on request from the corresponding author. The data are not publicly available due to privacy reasons.

## References

[B1-behavsci-15-00210] Afaq A., Gaur L., Singh G., Dhir A. (2023). COVID-19: Transforming air passengers’ behaviour and reshaping their expectations towards the airline industry. Tourism Recreation Research.

[B2-behavsci-15-00210] Amankwah-Amoah J. (2021). COVID-19 pandemic and innovation activities in the global airline industry: A review. Environment International.

[B3-behavsci-15-00210] Atasoy B., Türkay O., Şengül S. (2022). Strategic responses of chain hotels to COVID-19 from a situational crisis communication theory perspective. Journal of Hospitality and Tourism Insights.

[B4-behavsci-15-00210] Belhadi A., Kamble S., Jabbour C. J. C., Gunasekaran A., Ndubisi N. O., Venkatesh M. (2021). Manufacturing and service supply chain resilience to the COVID-19 outbreak: Lessons learned from the automobile and airline industries. Technological Forecasting and Social Change.

[B5-behavsci-15-00210] Byrnes K. P., Rhoades D. L., Williams M. J., Arnaud A. U., Schneider A. H. (2022). The effect of a safety crisis on safety culture and safety climate: The resilience of a flight training organization during COVID-19. Transport Policy.

[B6-behavsci-15-00210] Cho V., Lam W. (2021). The power of LinkedIn: How LinkedIn enables professionals to leave their organizations for professional advancement. Internet Research.

[B7-behavsci-15-00210] Coombs W. T. (2007). Protecting organization reputations during a crisis: The development and application of situational crisis communication theory. Corporate Reputation Review.

[B8-behavsci-15-00210] Eriksson M. (2018). Lessons for crisis communication on social media: A systematic review of what research tells the practice. International Journal of Strategic Communication.

[B9-behavsci-15-00210] Fox News (2021). Family kicked off Spirit flight for toddler not wearing mask, mom says.

[B10-behavsci-15-00210] Garaus M., Hudáková M. (2022). The impact of the COVID-19 pandemic on tourists’ air travel intentions: The role of perceived health risk and trust in the airline. Journal of Air Transport Management.

[B11-behavsci-15-00210] Handmer J., Dovers S. (2012). The handbook of disaster and emergency policies and institutions.

[B12-behavsci-15-00210] Hassankhani M., Alidadi M., Sharifi A., Azhdari A. (2021). Smart city and crisis management: Lessons for the COVID-19 pandemic. International Journal of Environmental Research and Public Health.

[B13-behavsci-15-00210] Hillson D. (2023). The risk management handbook: A practical guide to managing the multiple dimensions of risk.

[B14-behavsci-15-00210] Kaffash S., Khezrimotlagh D. (2023). US network and low-cost carriers’ performance in response to COVID-19: Strictness of government policies and passengers’ panic. Research in Transportation Business & Management.

[B15-behavsci-15-00210] Kasperson R. E., Renn O., Slovic P., Brown H. S., Emel J., Goble R., Kasperson J. X., Ratick S. (1988). The social amplification of risk: A conceptual framework. Risk Analysis.

[B16-behavsci-15-00210] Kaushal V., Srivastava S. (2021). Hospitality and tourism industry amid COVID-19 pandemic: Perspectives on challenges and learnings from India. International Journal of Hospitality Management.

[B17-behavsci-15-00210] Kwok L., Lee J., Han S. H. (2022). Crisis communication on social media: What types of COVID-19 messages get the attention?. Cornell Hospitality Quarterly.

[B18-behavsci-15-00210] Liu B. F., Fowler B. M., Roberts H. A., Herovic E. (2018). Keeping hospitals operating during disasters through crisis communication preparedness. Public Relations Review.

[B19-behavsci-15-00210] Manuel T., Herron T. L. (2020). An ethical perspective of business CSR and the COVID-19 pandemic. Society and Business Review.

[B20-behavsci-15-00210] Obembe D., Kolade O., Obembe F., Owoseni A., Mafimisebi O. (2021). COVID-19 and the tourism industry: An early stage sentiment analysis of the impact of social media and stakeholder communication. International Journal of Information Management Data Insights.

[B21-behavsci-15-00210] Ou J., Wong I. A. (2021). Strategic crisis response through changing message frames: A case of airline corporations. Current Issues in Tourism.

[B22-behavsci-15-00210] Pan J. Y., Liu D. (2022). Mask-wearing intentions on airplanes during COVID-19–Application of theory of planned behavior model. Transport Policy.

[B23-behavsci-15-00210] Prados-Peña M. B., Crespo-Almendros E., Porcu L. (2022). COVID-19 and social media communication strategies: A comparative study of the effectiveness of Facebook posts during the lockdown and the “new normal” in the airline industry. Journal of Air Transport Management.

[B24-behavsci-15-00210] Quan L., Al-Ansi A., Han H. (2022). Assessing customer financial risk perception and attitude in the hotel industry: Exploring the role of protective measures against COVID-19. International Journal of Hospitality Management.

[B25-behavsci-15-00210] Scheiwiller S., Zizka L. (2021). Strategic responses by European airlines to the COVID-19 pandemic: A soft landing or a turbulent ride?. Journal of Air Transport Management.

[B26-behavsci-15-00210] Schoofs L., Fannes G., Claeys A. S. (2022). Empathy as a main ingredient of impactful crisis communication: The perspectives of crisis communication practitioners. Public Relations Review.

[B27-behavsci-15-00210] Sharma G. D., Kraus S., Srivastava M., Chopra R., Kallmuenzer A. (2022). The changing role of innovation for crisis management in times of COVID-19: An integrative literature review. Journal of Innovation & Knowledge.

[B28-behavsci-15-00210] Shukla B., Sufi T., Joshi M., Sujatha R. (2023). Leadership challenges for Indian hospitality industry during COVID-19 pandemic. Journal of Hospitality and Tourism Insights.

[B29-behavsci-15-00210] Statista (2024). Total number of passengers carried by spirit airlines from 2003 to 2023.

[B30-behavsci-15-00210] Suk M., Kim W. (2021). COVID-19 and the airline industry: Crisis management and resilience. Tourism Review.

[B31-behavsci-15-00210] Thakur R., Hale D. (2022). Strategic crisis response: Managerial implications and direction for recovery and survival. Journal of Business & Industrial Marketing.

[B32-behavsci-15-00210] Thomsen S. R. (2023). “Not the company we thought it was.” Southwest Airlines’ attempt at image repair during its October 2021 flight cancellation crisis. Public Relations Review.

[B33-behavsci-15-00210] Varma T. M. (2021). Responsible leadership and reputation management during a crisis: The cases of Delta and United Airlines. Journal of Business Ethics.

[B34-behavsci-15-00210] Wang L., Schuetz C. G., Cai D. (2021). Choosing response strategies in social media crisis communication: An evolutionary game theory perspective. Information & Management.

[B35-behavsci-15-00210] Wilbur D., Cameron G. T. (2020). Theory meets practice: Updating the contingency theory of conflict management with Insights from an Adroit Practitioner. Romanian Journal of Communication and Public Relations.

[B36-behavsci-15-00210] Williams R. D., Dumas C., Ogden L., Flanagan J., Porwol L. (2024). Virtual reality training for crisis communication: Fostering empathy, confidence, and de-escalation skills in library and information science graduate students. Library & Information Science Research.

